# Adherence to guidelines for the prescription of secondary prevention medication at hospital discharge after acute coronary syndrome: a multicentre study

**DOI:** 10.1007/s12471-015-0664-y

**Published:** 2015-03-10

**Authors:** J. Tra, I. van der Wulp, Y. Appelman, M.C. de Bruijne, C. Wagner

**Affiliations:** 1 Department of Public and Occupational health, EMGO+/VU University medical center, van der Boechorststraat 7, 1081 BT Amsterdam, The Netherlands; 2 Department of Cardiology, VU University medical center, Amsterdam, The Netherlands; 3 The Netherlands Institute of Health Services Research (NIVEL), Utrecht, The Netherlands

**Keywords:** Acute coronary syndrome, Secondary prevention, Guideline adherence, Quality indicators, Acute coronary syndrome/drug therapy, Patient discharge

## Abstract

**Background:**

The prescription of guideline-recommended medication for secondary prevention after acute coronary syndrome has been suboptimal in the past. In the present study, guideline adherence and associated patient, care and hospital characteristics at hospital discharge after acute coronary syndrome were studied.

**Methods:**

Charts of patients with acute coronary syndrome discharged from 13 Dutch hospitals in 2012 were reviewed. Guideline adherence was defined as the prescription of acetylsalicylic acid, P2Y_12_ receptor inhibitor, statin, beta-blocker and angiotensin-converting enzyme (ACE) inhibitor at discharge, or a documented contraindication. Associated characteristics were identified by means of generalized linear mixed models for binary outcomes.

**Results:**

In total, 2471 patients were included. Complete guideline adherence was achieved in 69.1 % of the patients, ranging from 42.1 to 87.0 % between hospitals. The ACE inhibitor was most often missing (21.2 %). Patients with non-ST-segment elevation myocardial infarction or unstable angina, patients with a history of coronary artery bypass grafting or elderly women were less likely to be discharged with the guideline-recommended medication.

**Conclusions:**

Guideline adherence for secondary prevention medication following acute coronary syndrome was substantial; however, variation between hospitals and patient groups was found. Efforts to increase guideline adherence can focus on underperforming hospitals and undertreated patient groups.

## Background

In recent years, the in-hospital survival rates of patients with an acute coronary syndrome (ACS) have increased [1], yet patients with a history of ACS are at higher risk of adverse cardiac outcomes in the future [2]. As a result, discharge and post-discharge management, comprising referral to a cardiac rehabilitation program and the prescription of secondary prevention medication [3, 4], have become more important in ACS care.

Prescribing medication for secondary prevention of adverse cardiac outcomes after discharge from the hospital is recommended by the European Society of Cardiology guidelines on the management of ACS. This medication comprises a combination of acetylsalicylic acid, P2Y_12_ receptor inhibitor, statin, beta-blocker and angiotensin-converting enzyme (ACE) inhibitor [3, 4] However, previous studies have identified non-adherence to these guideline recommendations in several patient groups [5, 6]. As a result, these patients have a higher but potentially preventable risk of adverse outcomes after discharge [7].

Monitoring and improving guideline adherence for secondary prevention medication at hospital discharge has the potential to improve the quality of care and further reduce adverse outcomes in patients with ACS [8, 9]. This was recognised by Dutch cardiology care providers, who included a focus on discharge medication in a national quality improvement program [10]. In this study we investigated guideline adherence and associated patient, care and hospital characteristics for secondary prevention medication at discharge from the hospital for patients with ACS in the Netherlands during implementation of a nationwide quality improvement program.

## Methods

A detailed description of the study design, methods and the quality improvement program has previously been published [11].

### Design

The study was conducted in a cross-sectional design.

### Setting and Inclusion

In 2012, 91 hospitals provided ACS care in the Netherlands. From this pool, 13 hospitals were selected by means of a multistage random sampling procedure to participate in the evaluation of the national quality improvement program.

Potentially eligible study charts were selected from the hospitals’ financial system codes for ST-segment elevation myocardial infarction (STEMI), non-ST-segment elevation myocardial infarction (NSTEMI) and unstable angina (UA). All patients discharged in 2012 with a diagnosis of ACS (as confirmed in the discharge letter) were considered for inclusion. Charts of patients transferred to another hospital or department for further evaluation or treatment, patients who died during hospital admission, who received palliative care, who left the hospital against medical advice or who had no information about the prescribed medication at discharge in their chart were excluded.

### Data collection and processing

Data were collected by means of retrospective chart review. The chart reviewers visited the participating hospitals monthly. When the number of charts exceeded the screening capacity, charts were selected per month in chronological order of discharge until the screening capacity limit was reached.

In this study, guideline adherence was defined as the prescription of acetylsalicylic acid, P2Y_12_ receptor inhibitor, statin, beta-blocker and ACE inhibitor at discharge, or a documented contraindication or other motivation for not prescribing these medicines. From the charts, information related to the prescription of these five medicines was abstracted. In case one or more medicines were not prescribed, documented contraindications as reported in an annually updated Dutch database of pharmacotherapy [12], or as motivated by the treating physician were retrieved (e.g. the prescription of anticoagulants instead of acetylsalicylic acid). The full list of contraindications was reported previously [11]. Additionally, patient, care and hospital characteristics (*n* = 40), e.g. age, sex, cardiac medical history, risk factors, resuscitation and discharge diagnosis were recorded. Hospitals were characterised by type (academic, tertiary teaching or general) and presence of percutaneous coronary intervention (PCI) and/or coronary artery bypass grafting (CABG) facilities (yes/no).

A sample of the charts (*n* = 149 (6.0 %)) was screened by two chart reviewers independently and the percentage of agreement between the reviewers was calculated. The results were satisfactory, with all 40 variables above 85 % agreement indicating good to excellent data reliability.

### Missing data

In total, 0.82 % of the data were missing, ranging per variable from 0.04 % (date of discharge) to 2.5 % (heart failure or arrival). Little’s test [13] was non-significant (*p* = 0.57) and missing value analyses showed no relationship between the missing data and the complete data, indicating the missing data were missing completely at random [14]. Therefore missing data were imputed by means of full conditional specification using the imputation procedure in IBM SPSS (Version 20 for Windows). As a sensitivity analysis, the results of the final analysis were compared with the results of a full case analysis to determine the accuracy of the imputation procedure.

### Analysis

Characteristics of the study population, participating hospitals and guideline adherence were determined by means of descriptive statistics. Associations of predictor variables with guideline adherence (complete adherence vs incomplete adherence) were studied by means of generalized linear mixed models for binary outcomes. To correct for clustering of patients within hospitals, hospital was entered as random effect in the analyses.

The associations of the predictor variables with guideline adherence were tested in univariate models (Table 1). All predictor variables with a significant association (*p* ≤ 0.05) were added to a multivariable model. To account for collinearity, all predictor variables without a significant association in the univariate analyses were added to the multivariable model one by one. In case of a significant improvement of the model fit (*p* ≤ 0.05), they were added to the multivariable model. Additionally, several potential interactions between variables were tested in the multivariable model: age with treatment, discharge diagnosis and sex; and discharge diagnosis with treatment and sex. In case of a significant improvement of the model fit (*p* ≤ 0.05), the interactions were added to the multivariable model. From this model, the fixed effects were presented as odds ratios (OR) with 95 % confidence intervals (CI).

**Table 1:** Associations of patient, arrival, discharge and hospital characteristics with prescription of discharge medication in univariable generalized linear mixed models (*N* = 2471)

Variable	*n* (%)	Incomplete guideline adherence (*n* = 763)	Guideline adherence (*n* = 1708)	*P*-value^a^
Discharge diagnosis				***< 0.001
STEMI	910 (36.8 %)	161 (21.1 %)	749 (43.9 %)	
NSTEMI	987 (39.9 %)	310 (40.6 %)	677 (39.6 %)	
UA	574 (23.2 %)	292 (38.3 %)	282 (16.5 %)	
Type of treatment				***< 0.001
Medication	793 (32.1 %)	355 (46.5 %)	438 (25.6 %)	
PCI	1552 (62.8 %)	360 (47.2 %)	1192 (69.8 %)	
CABG	126 (5.1 %)	48 (6.3 %)	78 (4.6 %)	
Age in years (mean, 95 % CI)	66.9 (66.4–67.4)	65.9 (65.3–66.5)	69.1 (68.1–70.0)	***< 0.001
Female	801 (32.4 %)	287 (37.6 %)	514 (30.1 %)	***< 0.001
Admission				
Resuscitation	100 (4.0 %)	9 (1.2 %)	91 (5.3 %)	***< 0.001
Heart failure on arrival	138 (5.6 %)	35 (4.6 %)	103 (6.0 %)	0.29
Cardiogenic shock on arrival	35 (1.4 %)	11 (1.4 %)	24 (1.4 %)	0.79
Transportation from another hospital	394 (15.9 %)	97 (12.7 %)	297 (17.4 %)	**0.002
Discharge				
Month of discharge	N/A	N/A	N/A	0.22
Weekend discharge	724 (29.3 %)	226 (29.6 %)	498 (29.2 %)	0.85
Length of stay (median days, 1st-3rd quartile)^b^	5 (3–7)	5 (4–7)	4 (3–6)	***< 0.001
Risk factors				
Diabetes mellitus	545 (22.1 %)	118 (15.5 %)	427 (25.0 %)	***< 0.001
Hypertension	1204 (48.7 %)	389 (51.0 %)	815 (47.7 %)	0.10
Kidney failure	111 (4.5 %)	22 (2.9 %)	89 (5.2 %)	*0.02
Chronic heart failure	103 (4.2 %)	31 (4.1 %)	72 (4.2 %)	0.61
Positive family history	910 (36.8 %)	281 (36.8 %)	629 (36.8 %)	0.64
Coronary stenosis	205 (8.3 %)	79 (10.4 %)	126 (7.4 %)	**0.003
Hyperlipidaemia^c^	1208 (48.9 %)	375 (49.1 %)	833 (48.8 %)	0.61
Obesity (BMI > 30 kg/m^2^)	270 (10.9 %)	80 (10.5 %)	190 (11.1 %)	0.64
Smoker	739 (29.9 %)	184 (24.1 %)	555 (32.5 %)	***< 0.001
Former smoker	452 (18.3 %)	138 (18.1 %)	314 (18.4 %)	0.99
Cardiac medical history				
Angina pectoris	442 (17.9 %)	167 (21.9 %)	275 (16.1 %)	**0.001
Peripheral vascular disease	159 (6.4 %)	50 (6.6 %)	109 (6.4 %)	0.69
Coronary artery disease	264 (10.7 %)	96 (12.6 %)	168 (9.8 %)	*0.03
Prior MI	574 (23.2 %)	189 (24.8 %)	385 (22.5 %)	0.21
Prior PCI	592 (24.0 %)	206 (27.0 %)	386 (22.6 %)	*0.02
Prior CABG	303 (12.3 %)	125 (16.4 %)	178 (10.4 %)	***< 0.001
Recent PCI, CABG or MI (< 6 months before admission)	142 (5.7 %)	42 (5.5 %)	100 (5.9 %)	0.51
Hospital characteristics				
Type of hospital				0.26
General hospital (*n* = 4)	648 (26.2 %)	245 (32.1 %)	403 (23.6 %)	
Tertiary teaching hospital (*n* = 7)	1426 (57.7 %)	430 (56.4 %)	996 (58.3 %)	
Academic hospital (*n* = 2)	397 (16.1 %)	88 (11.5 %)	309 (18.1 %)	
Treated in hospital with PCI facilities (*n* = 7)	1512 (61.2 %)	448 (58.7 %)	1064 (62.3 %)	0.73
Treated in hospital with CABG facilities (*n* = 5)	958 (38.8 %)	290 (38.0 %)	668 (39.1 %)	0.87

*significant at ≤ 0.05 level; **significant at ≤ 0.01 level; ***significant at ≤ 0.001 level

*STEMI* ST-segment elevation myocardial infarction, *NSTEMI* non-ST-segment elevation myocardial infarction, *UA* unstable angina pectoris, *PCI* percutaneous coronary intervention, *CABG* coronary artery bypass grafting, *CI* confidence interval, *N/A* not applicable, *BMI* body mass index, *MI* myocardial infarction

^a^P-values are calculated using the Wald statistic, comparing the model fit of a generalized linear mixed model with and without the variable, corrected for clustering of patients in hospitals

^b^Length of stay was log-transformed after careful consideration of the residuals of a model without random intercept

^c^Hyperlipidaemia was defined as described in patients history or statin use before admission

The data were analysed in R (version 3.0.2 for Windows) using the *lme4* package.

### Ethical approval

The study protocol was approved by the medical ethics review committee of the VU University medical center.

## Results

### Selection of patient charts

In total, 3427 charts of patients with a confirmed discharge diagnosis of ACS in 2012 were screened. Of these, 876 patients (26.6 %) were transferred to another hospital or department for further evaluation or treatment, 56 (1.6 %) died during admission, information concerning discharge medication was missing for 14 patients (0.4 %), 6 (0.2 %) left the hospital against medical advice and 4 (0.1 %) received palliative care. After exclusion of these charts, 2471 patients were eligible for further analyses. Their mean age was 66.9 years and the majority were male (67.6 %) (Table 1).

### Guideline adherence

**Fig. 1:**
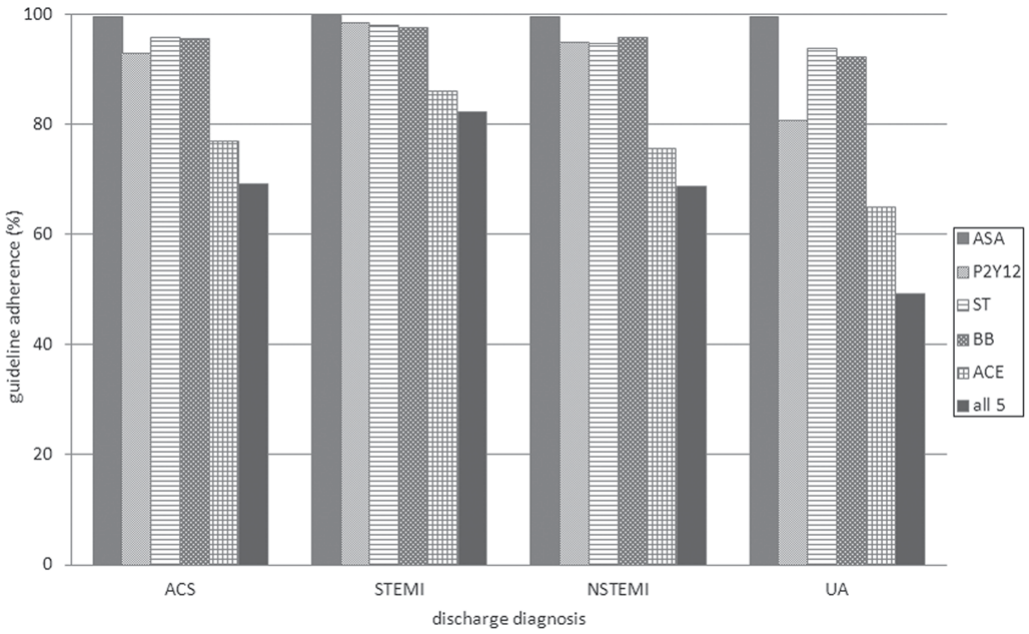
Guideline adherence (%) per medicine per discharge diagnosis. *ASA* acetylsalicylic acid, *P2Y*_12_ P2Y_12_ receptor inhibitor, *ST* statin, *BB* beta-blocker, *ACE* angiotensin-converting enzyme inhibitor, *ACS* acute coronary syndrome, *STEMI*ST-elevation myocardial infarction, *NSTEMI* non-ST-elevation myocardial infarction, *UA* unstable angina

Overall, 49.1 % of the patients were prescribed all five guideline-recommended medicines at discharge from the hospital, while an additional 20.0 % had contraindications documented for the medicines that were not prescribed. Consequently, the complete guideline adherence for the combination of the five medicines was 69.1 %. Guideline adherence for the individual medicines ranged between 99.6 % for acetylsalicylic acid and 76.8 % for the ACE inhibitor. Complete guideline adherence for the combination of the five medicines ranged from 42.1 to 87.0 % between hospitals. Prescription rates and guideline adherence are presented in Table 2 and Fig. 1.

**Table 2:** Prescription patterns for the five medicines for secondary prevention (*N* = 2471)

Drug type	Prescriptions *n* (%)	Range (% in lowest – highest scoring hospital)	Guideline adherence^a^ *n* (%)	Range (% in lowest – highest scoring hospital)
Acetylsalicylic acid	2271 (91.9 %)	86.6–97.2 %	2460 (99.6 %)	98.6–100 %
P2Y_12_ receptor inhibitor	2189 (88.6 %)	70.3–95.9 %	2293 (92.8 %)	75.7–98.6 %
Statin	2294 (92.8 %)	81.1–97.4 %	2363 (95.6 %)	83.8–98.9 %
Beta-blocker	2220 (89.8 %)	83.5–99.0 %	2360 (95.5 %)	90.5–99.0 %
ACE inhibitor	1603 (64.9 %)	47.5–74.5 %	1898 (76.8 %)	57.9–93.1 %
All 5 medicines	1214 (49.1 %)	28.2–59.0 %	1708 (69.1 %)	42.1–87.0 %
4 out of 5 medicines	2068 (83.7 %)	67.8–91.0 %	2297 (93.0 %)	78.4–99.0 %

*ACE* angiotensin-converting enzyme, *STEMI* ST-segment elevation myocardial infarction, *NSTEMI* non-ST-segment elevation myocardial infarction, *UA* unstable angina pectoris

^a^Guideline adherence refers to either prescription of the medicine or documentation of a contraindication

### Factors associated with (in)complete guideline adherence

In univariate generalized linear mixed model analyses, discharge diagnosis, type of treatment, age, sex, resuscitation, transport from another hospital and length of stay were significantly associated with the probability of guideline adherence (Table 1). Additionally, the risk factors diabetes mellitus, kidney failure, a prior detected coronary stenosis, current smoking, and a medical history of angina pectoris, coronary artery disease, prior PCI or prior CABG were associated with the probability of guideline adherence. These were entered in a multivariable model. The variable ‘recent PCI, CABG or myocardial infarction (< 6 months before admission)’ significantly improved the multivariable model fit and was therefore subsequently added to the model secondarily. In addition, an interaction between age and sex was added to the multivariable model. No significant associations between hospital characteristics and guideline adherence were found.

In the final model, patients with NSTEMI or UA compared with patients with STEMI, and patients who had a prior CABG were less likely to receive the guideline-recommended medication at discharge (Table 3). Further, adherence was higher for patients who were treated with PCI compared with patients who received pharmacological or CABG treatment, who had diabetes mellitus or kidney failure, who were resuscitated on admission, who had longer lengths of hospital stay, or who had a recent PCI, CABG or myocardial infarction (< 6 months before admission). Additionally, the effect of age differed between men and women, i.e. the medication was less likely completely according to the guidelines for older women compared with older men.

**Table 3:** Associations of patient, arrival, discharge and hospital characteristics with prescription of secondary prevention medication in the multivariable generalized linear mixed model

Variable	OR (95 % CI)	*P*-value
Discharge diagnosis		
STEMI (intercept)	N/A	0.37
NSTEMI	0.64 (0.49–0.82)	***< 0.001
UA	0.29 (0.21–0.39)	***< 0.001
Female patient	4.09 (1.32–12.7)	**< 0.01
Coronary artery disease	1.13 (0.79–1.61)	0.51
Angina pectoris	1.04 (0.79–1.38)	0.76
Prior PCI	0.98 (0.76–1.26)	0.87
Prior CABG	0.70 (0.51–0.95)	*0.02
Diabetes mellitus	2.67 (2.06–3.45)	***< 0.001
Kidney failure	2.10 (1.23–3.57)	*0.007
Smoker	1.19 (0.94–1.50)	0.16
Coronary stenosis	0.82 (0.56–1.20)	0.30
Resuscitation	2.65 (1.29–5.44)	**0.008
Transportation from another hospital	0.81 (0.60–1.10)	0.18
Age	0.97 (0.96–0.99)	***< 0.001
Length of stay^a^	1.43 (1.19–1.72)	***< 0.001
PCI	2.05 (1.63–2.59)	***< 0.001
CABG	0.98 (0.63–1.55)	0.94
Recent PCI, CABG or MI (< 6 months before admission)	1.91 (1.23–2.95)	**0.004
Interaction between Age and Sex	1.02 (1.00–1.04)	**0.003

*significant at ≤ 0.05 level; **significant at ≤ 0.01 level; ***significant at ≤ 0.001 level

*STEMI* ST-segment elevation myocardial infarction, *N/A* not applicable, *NSTEMI* non-ST-segment elevation myocardial infarction, *UA* unstable angina pectoris, *PCI* percutaneous coronary intervention, *CABG* coronary artery bypass grafting, *MI* myocardial infarction

^a^Length of stay was log-transformed after careful consideration of the residuals of a model without random intercept

### Sensitivity analysis

As a sensitivity analysis the current model was compared with a full case analysis model (*n* = 2253). No differences were found in variable selection or significant associations, indicating a reliable imputation procedure.

## Discussion

In this multicentre study, guideline adherence for secondary prevention medication prescription at hospital discharge for patients with ACS was investigated. Complete guideline adherence was 69.1 %, with the highest adherence for acetylsalicylic acid and the lowest for the ACE inhibitor. Several patient and care characteristics were significantly associated with guideline adherence, while hospital characteristics were not.

The level of complete guideline adherence found in this study was comparable with another Dutch study in which 65.2 % of the patients with ACS were discharged with the recommended secondary prevention discharge medication [15]. Also, in accordance with other studies, guideline adherence for the recommended discharge medication was lowest for the ACE inhibitor [16]. This finding may be explained by the recommendation to prescribe the ACE inhibitor to all patients with ACS, but an exception may be made for normotensive patients without heart failure, left ventricular (LV) dysfunction or diabetes mellitus [4]. Furthermore, an angiotensin-II-receptor inhibitor can be prescribed as an alternative for the ACE inhibitor, although the ACE inhibitor is the primary choice, as previous studies showed superiority in reducing adverse outcomes [17]. Therefore, prescription of an angiotensin-II-receptor inhibitor was not abstracted from the charts in this study.

Although there was substantial variation in guideline adherence between hospitals, this could not be explained by the hospitals’ characteristics (presence of intervention facilities and type of hospital). A recent study found that hospitals with interventional facilities treated more patients according to the guidelines [15]. The different findings in this study might be explained by the use of a statistical correction for clustering of patients in hospitals, thereby reducing the effective sample size [18]. When no statistical correction for clustering is used, the hospital characteristics are attributed to individual patients (*n* = 2471) instead of hospitals (*n* = 13), resulting in spurious significant results.

The odds of complete guideline adherence were lowest for patients with UA, intermediate for NSTEMI patients and highest for patients with STEMI. This finding confirms the results of previous studies [19, 20]. The difference in guideline adherence between STEMI and NSTEMI/UA might be explained by small differences in the European Society of Cardiology guidelines for the management of ACS patients with and without ST-segment elevation. One difference is the recommendation to prescribe a beta-blocker to all patients with STEMI, while only to patients with NSTEMI/UA who have LV dysfunction. However, this difference in the guidelines does not explain the differences found in the present study, as the differences between STEMI and NSTEMI/UA were mostly caused by lower guideline adherence for the ACE inhibitor and the P2Y_12_ inhibitor. Moreover, the high prescription rates of beta-blockers in NSTEMI and UA patients might indicate overmedication or adherence, potentially caused by adherence to previous guidelines [21] as guideline adoption takes time.

Interestingly, the negative association of age with guideline adherence was stronger in women than in men. This finding is worrisome, as the most recent European guidelines recommend managing both genders in a similar fashion [22]. To our knowledge, there is limited information on the impact of sex and age on the decision of physicians to prescribe secondary prevention medication after acute coronary syndrome. Therefore, additional research is required to identify potential (reasons for) treatment biases of physicians towards treatment of elderly women.

Since the estimated guideline adherence for secondary prevention medication after ACS was substantial, it would be recommended to focus future quality improvement efforts on reducing variation between patient groups and between hospitals. Several interventions exist for improving guideline adherence, e.g. feedback of performance [23], continuing education [24] or integrated care [25]. These interventions can focus on undertreated patient groups, e.g. facilitating educational meetings for cardiology care providers about the treatment of elderly women with ACS.

## Limitations

Several limitations of this study might influence interpretation of its results.

Having no information on one of the medicines prescribed at discharge in the patient chart can be the result of no prescription, or no documentation of the prescription, contraindication or another reason for not prescribing the medicine (e.g. preserved LV function for the ACE inhibitor). As the charts were not screened by physicians, implicit decisions could not be included in the chart review. Instead, a standard list of contraindications was used, thus minimising inter-reviewer variation. Consequently, the rates of guideline adherence in this study might differ slightly from real-life guideline adherence and are therefore only an estimate. An additional limitation relating to documentation is the selection of patients by means of the hospital billing systems with manual review of the discharge diagnosis in the chart. Potentially some ACS patients were missed who received a billing code not related to ACS or with a different discharge diagnosis documented in their chart, which could limit the external validity of the findings in this study.

Another limitation of this study was that the potential prescription of suboptimal doses of medicines was counted as adhering to the guidelines [26] As optimal doses tend to differ between patients, and the optimal dose can often not be prescribed at discharge, it was not possible to incorporate doses in this study. Counting suboptimal doses as guideline adherence may have led to an overestimation of the quality of care in this study. In addition, prescribing medicines that are not indicated can also be considered to be incomplete guideline adherence. However, as the charts were not screened by physicians, this could not be taken into account in this study.

Prescription behaviour of individual physicians can be an additional source of variation in guideline adherence [27]; however, in this study it was not feasible to identify the physician responsible for medication prescription at discharge from the charts.

As a result of the large number of patients in PCI centres, the screening capacity limited the number of included patient charts per month. However, this should not have affected the results in our study since patients were selected in chronological order of discharge and there was no difference in guideline adherence between hospitals with and without PCI and/or CABG facilities.

Finally, participation in a national quality improvement program and the monthly measurements might have overestimated guideline adherence. However, the effect of this program on guideline adherence is expected to be limited as no significant association between month of discharge and guideline adherence was found.

## Conclusion

Guideline adherence concerning the prescription of discharge medication in the Netherlands is substantial though differs between hospitals and patient groups. Efforts to further improve guideline adherence can be targeted on those patient groups who receive suboptimal treatment at discharge from the hospital, e.g. elderly women and patients with NSTEMI or UA.
